# Housing Australian Children: A Snapshot of Health Inequities in the First 2000 Days

**DOI:** 10.1007/s11524-024-00925-0

**Published:** 2024-11-01

**Authors:** Yuxi Li, Ankur Singh, Rebecca Bentley

**Affiliations:** 1 Centre for Health Policy, Melbourne School of Population and Global Health, University of Melbourne, 207 Bouverie St, Carlton VIC, 3053 Australia; 2 Centre for Epidemiology and Biostatistics, School of Population and Global Health, University of Melbourne, Melbourne, Australia

**Keywords:** Housing, Child health, Early life, Health inequities

## Abstract

Housing is a critical social determinant of children’s health. While individual pathways between housing and health are well evidenced, there has been less attention on the co-occurrence of housing disadvantages. We aim to identify typologies of children’s housing disadvantage and describe the health inequities they generate. A cross-sectional latent class analysis (LCA) was conducted on 4355 Australian children aged 4 to 5 years who participated in the Longitudinal Study of Australian Children. Nine binary indicators—dwelling tenure, external condition, affordability, stability, crowding, cleanliness, fuel poverty, and noise exposure—were used to generate latent housing typologies. Generalized linear models with robust standard errors described disparities in quality of life, injury, disability, and health service use across these housing typologies. Four distinct housing typologies were identified in this cohort: “good condition, affordable, and secure” (60%); “insecure” (11%); “crowded” (24%); and “unsuitable” (5%). Unsuitable housing, characterized by crowding, poor external conditions, and noise exposure, was more likely to be occupied by single-parent families, low-income households, and be headed by parents with low levels of education. Children in unsuitable housing lagged on quality of life indicators at preschool age (e.g., by 8.0 points on emotional functioning, 95% CI − 10.6, − 5.5), and underutilized primary healthcare services (e.g., prevalence ratio 0.76 for GP services, 95% CI 0.67, 0.87), compared to children in good quality housing. This finding supports the case for early intervention strategies that account for children’s housing circumstances.

**Supplementary Information:**

The online version contains supplementary material available at 10.1007/s11524-024-00925-0.

## Introduction

Housing is a critical determinant of children’s health and development [1]. Specific pathways have been identified between housing and the health of children, including the associations between housing insecurity and psychiatric disorders [2], poor quality housing and hospitalization for injury [3], and indoor mold exposure and asthma [4]. There is also a wealth of literature on developmental outcomes, such as frequent moves and socio-emotional development [5], unaffordable housing, and cognitive skills [6]. Moreover, housing disadvantage in childhood compounds the negative consequences for health and development of broader socio-economic disadvantage [7].

Two main challenges have hampered our understanding of housing disadvantage and its implications for children’s health and social outcomes. Previous research has largely focussed on the effect of singular dimensions of housing, especially physical housing conditions, and failed to capture the co-occurrence of multiple housing disadvantages, such as insecurity and unaffordability [8]. While this is important from an intervention perspective this does not reflect the housing realities of children where multiple disadvantages may co-occur. Additionally, there is a dearth of research on the consequences for health and well-being of exposure to housing disadvantage in the formative years of childhood—often identified as the first 2000 days of life. This period, representing conception to age 5 years, is increasingly recognized as a critical period affecting lifetime health, educational, and economic outcomes [9]. It has also been identified as a key window within which to break cycles of disadvantage that compound generational inequities in health [10].

We argue that examining the co-occurrence of housing experiences, or the ways housing attributes are clustered, allows us to identify children and families who are in disadvantaged housing circumstances that negatively impact their health and well-being. In conceptualizing housing as a determinant of health, we draw upon three dimensions of housing identified by the WHO Housing and Health Guidelines [11] and the recently published healthy housing glossary [12]: affordability (e.g., housing affordability stress, fuel poverty), security (e.g., frequent moves, forced eviction), and suitability (e.g., housing conditions). We use latent class analysis (LCA) to describe the housing experiences of a nationally representative cohort of participants aged 4–5 years of age in the Longitudinal Study of Australian Children based on these available measures. Clusters were identified using all housing-related information in the dataset. We then describe the socio-demographic and health characteristics of children in each housing typology identified using the latent class approach. Our objective is to address the following research questions:

1. What types of housing disadvantages are experienced by Australian children?
2. Are there inequities in health and health service utilization related to these patterns of exposure?

## Methods

This study adheres to the Strengthening the Reporting of Observational Studies in Epidemiology (STROBE) reporting guidelines [13]. We also referred to the Guidelines for Reporting on Latent Trajectory Studies (GRoLTS) checklist where relevant to this cross-sectional analysis [14].

### Design, Setting, and Participants

The Longitudinal Study of Australian Children (LSAC) is a nationally representative, dual cohort cross-sequential study of children and families in Australia. Initiated in 2004 and followed up biennially, it collects information on child development and wellbeing over the life course in relation to topics such as housing, family, peers, education, health, and healthcare utilization [15]. For its Kindergarten cohort (K cohort), 4983 children born between March 1999 and February 2000 (aged 4 to 5 at Wave 1 data collection) were randomly selected from the Medicare database. Survey instruments were tested for reliability and validity [16], and details of the study design were described elsewhere [17]. We analyzed Wave 1 data of the K cohort. Participants with missing data on housing variables were excluded, leaving a final sample of 4355 children aged 4 to 5 years.

### Measures

#### Latent Class Indicators

All questions related to housing in LSAC were examined, and nine binary variables were used to identify latent classes. *Unaffordability* was indicated by parents paying more than 30% of income on housing costs when the household income is below 40% of the national income distribution(0, no; 1, yes). *Fuel poverty* was measured by parents indicating they have been unable to heat or cool their home (0, no; 1, yes). *Noise* was measured by interviewer-reported background noise (0, moderate or no background noise; 1, loud background noise). *External condition* was derived from interviewer-assessed external conditions of the dwelling (0, well-kept condition; 1, poor or badly deteriorated condition). *Housing tenure* was broadly grouped into two categories (0, homeownership; 1, renting). *Dwelling type* was categorized according to whether the family lives in the house or not (0, living in houses; 1, not living in houses (e.g., living in apartments and mobile homes)). *Cleanness* was measured by interviewer-observed clutters in visible rooms (0, uncluttered; 1, cluttered). *Crowding* was measured by the ratio of bedrooms to the number of people in the household. Drawing on previous Australian research on crowding, we determined households as crowded if the ratio is equal to or greater than two [18]. *Instability* was indicated by parents reporting moving three times or more since the birth of the child.

#### Sociodemographic Variables

The following self-reported sociodemographic variables were obtained from the Wave 1 survey: gender (1, male; 2, female); main language spoken at home (1, English; 2, others); maternal education (1, year 11 or below; 2, year 12 or certificate; 3, bachelor degree or above); single parenthood (0, no; 1, yes); low income (defined by income below the 40% of national distribution, 0, no; 1, yes); residing area remoteness (1, urban cities; 2, regional or remote area).

#### Health Variables

The 23-item pediatric quality of life inventory (PedsQL) was used to measure general health and development in children and young people [19]. It includes four constructs: physical functioning (8 items), emotional functioning (5 items), school functioning (5 items), and social functioning (5 items). The score of each construct ranges between 0 and 100, with higher scores indicating better quality of life. A change of 4.5 points on the PedsQL score is considered a clinically important difference [20].

We also assessed the prevalence of disability and injury. Disability (yes/no) was self-reported by mothers through a question “Does your child have any long-term limiting health conditions?,” and injury (yes/no) was also reported by mothers through a question “Was your child injured in the last 12 months?”.

For a subsample who indicated use of healthcare services in the last 12 months (*n* = 3375), we described the type of services used, including uses of GP services, use of maternal and health services (i.e., a primary health service for families with children from birth to school age, including 24-h phone line, nurse visits, and education programs), use of emergency wards, and hospital outpatient care.

### Statistical Analysis

Latent class analysis (LCA) is a statistical technique that seeks to identify an underlying categorical latent variable that divides a population into mutually exclusive and exhaustive subgroups [21]. The aim of LCA is to maximize homogeneity within subgroups and maximize heterogeneity between groups. We use this technique to identify subgroups of children experiencing similar housing disadvantages. The numbers of latent classes (i.e., housing typologies) were tested incrementally from 2 to 7. The decision on the best-fit model was made considering the Akaike information criteria (AIC), Bayesian information criteria (BIC), and interpretation of the typologies. Lower values of AIC and BIC indicate a better balance between goodness-of-fit and complexity [22]. Alternative indices, such as the adjusted BIC (aBIC), were not used given the adequacy of our sample size [23]. Individuals were then assigned to the typology in which they had the highest posterior probability. Then, we described and compared the social and economic characteristics of each typology by crosstabulation and chi-square tests.

To assess health inequities by each housing typology, we used generalized linear regression with robust standard errors. For continuous health outcomes (i.e., quality of life measures), linear regression with identity link was used; for binary health outcomes (i.e., injury, disability, and health services use), log-binomial regression was used [24]. We then plotted the posterior prevalence/score of health, developmental, and health service use outcomes by each housing typology, adjusting for children’s gender, maternal age, and sample weights. Analyses were done in STATA SE 17.0 (StataCorp, 2021).

### Role of Funding Source

The study funders had no role in the design and conduct of the study; collection, management, analysis, and interpretation of the data; preparation, review, or approval of the manuscript; or decision to submit the manuscript for publication.

## Results

Table 1 presents the sociodemographic characteristics and housing conditions of the 4355 preschool-aged Australian children included in this study. The majority of the children lived in urban areas (85%) and used English as the primary language at home (88%). Fourteen percent of the sample belonged to single-parent households, and 27% were from low-income families. Approximately 22% of the mothers had an education level of year 11 or below. A significant portion of the families (33%) had moved at least three times since the birth of the child, and more than a quarter (29%) were renters. Notably, about 25% of children were exposed to either noise pollution or poor dwelling conditions, and 8.5% of families reported experiencing housing affordability stress. Over 70% of the children faced multiple housing disadvantages. The sociodemographic profile of the excluded individuals (due to missing data on housing variables) was similar to the analytical sample. Excluded individuals had slightly higher proportions of low-income (30.6% vs. 27.3%) and lone-parent households (15.8% vs. 13.7%).

**Table 1:** Sample characteristics

	Sample (*n* = 4355)		Excluded individuals (*n* = 628)	
Sociodemographic groups	*n*	%	*n*	%
Sex				
Male	2198	50.5	338	53.8
Female	2157	49.5	290	46.2
Remoteness				
Major urban cities	3704	85.1	512	81.5
Regional or remote area	644	14.8	115	18.3
*Missing*	7	0.2	1	0.16
Language spoken at home				
English	3829	87.9	530	84.4
Others	526	12.1	98	15.6
Maternal education				
Year 11 or below	947	21.8	133	21.2
Year 12 or certificate	2157	49.5	295	47.0
Bachelor or above	1220	28.0	188	30.0
*Missing*	31	0.7	12	1.9
Low income				
No	3167	72.7	436	69.4
Yes	1188	27.3	192	30.6
Single parenthood				
No	3757	86.3	529	84.2
Yes	598	13.7	99	15.8
Housing disadvantage				
Moved at least 3 times since birth	1449	33.3		
Unaffordable housing	372	8.5		
Noise	1136	26.1		
Poor external condition	1222	28.1		
Renting	1260	28.9		
Fuel poverty	167	3.8		
Living in apartments	502	11.5		
Overcrowding	441	10.13		
Cluttered home	379	8.7		
Number of housing disadvantages				
1	1161	26.7		
2	1228	28.2		
3	918	21.1		
> = 4	1048	24.0		

### Latent Class Model (Objective 1)

Fit statistics of models with two through to seven latent classes are presented in Supplementary Table 1. AIC suggests a 7-class solution, and BIC suggests a 3-class or 4-class model. Although consensus has not been reached, recommendations have been made that BIC offers a better justification over AIC for the number of classes [25]. Entropy was also taken into consideration and a value closer to 1 (and over 0.7) indicates a good classification for individuals in the sample. After considering interpretability and model fit, we selected a four-class model as the final model.

**Fig. 1:**
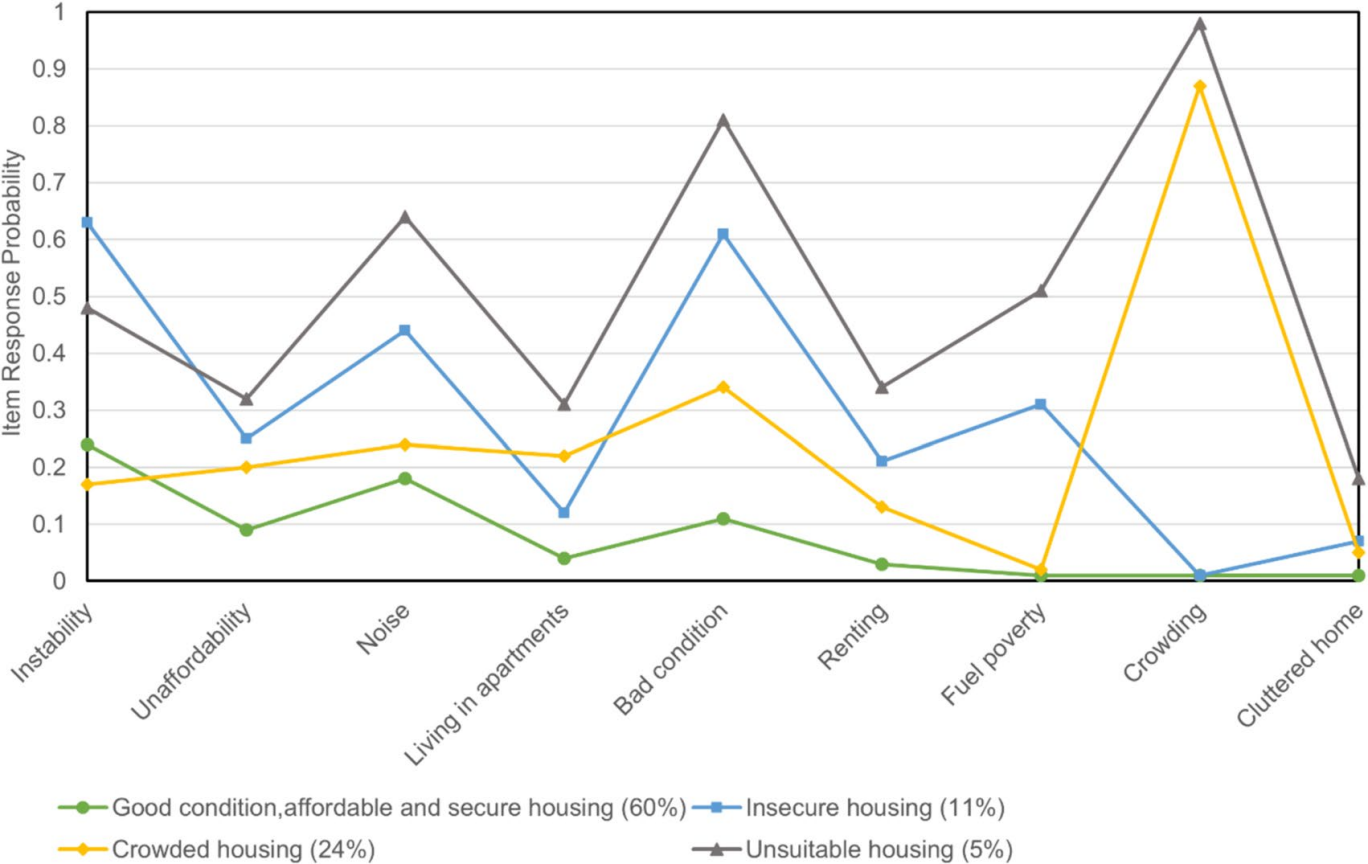
Item-response probabilities of housing variables

Item probabilities indicate the likelihood of individuals belonging to a certain typology reporting the housing disadvantage. For example, 87% of the individuals in typology 3 responded “yes” to overcrowding. Housing disadvantages that have either high (> 0.6) or low (< 0.4) item probabilities are characteristics of that class [26]. Using item-response probabilities, we interpreted the four unordered typologies as shown in Fig. 1:

Typology 1 (*n* = 2618, 60%) is characterized by attributes such as homeownership, well-kept external conditions, and absence of overcrowding. As none of the item probabilities was over 0.6, it represents a baseline against which the disadvantages of other classes are contrasted, highlighting the absence of immediate housing-related stressors. This typology was labeled “good condition, affordable, and secure.”

Typology 2 (*n* = 465, 11%) is characterized by indicators such as frequent relocations and renting instead of owning property. This category reflects a dimension of housing insecurity that can induce stress and anxiety, thereby impacting children’s sense of security and belonging. The label “insecure” underscores the psychosocial impact of unstable housing, focusing on the emotional and psychological consequences rather than solely on physical housing conditions.

Typology 3 (*n* = 1063, 24%) is marked by the high probability (87%) of its members reporting crowded homes. We called it “crowded” to capture the direct physical and indirect psychological impacts of living in constrained spaces.

Typology 4 (*n* = 209, 5%) combines several adverse conditions—poor external conditions, noise exposure, and crowding—suggesting disadvantage. The designation “unsuitable” reflects a broader inadequacy of the living environment, affecting not only health through physical pathways (e.g., exposure to harmful environmental factors) but also through psychological stress and social stigma associated with poor housing.

Table 2 presents the sociodemographic characteristics of children in each housing typology. Maternal education, income, and single parenthood were strongly associated with housing typologies. Children in the “unsuitable” group were more likely to come from low-income, single-parent households and have mothers with lower educational attainment compared to those in the “good condition” group.

**Table 2:** Sociodemographic characteristics of each housing typology (*N* = 4355)s

	Typology 1: Good housing (*n* = 2618)	Typology 2: Insecure housing (*n* = 465)	Typology 3: Crowded housing (*n* = 1063)	Typology 4: Unsuitable housing (*n* = 209)	
Sociodemographic groups	*n* (%)	*n* (%)	*n* (%)	*n* (%)	*p*-value of *χ*2 test
Sex					< 0.001
Male	1338 (51.1%)	228 (49%)	524 (49.3%)	108 (51.7%)	
Female	1280 (48.9%)	237 (51%)	539 (50.7%)	101 (48.3%)	
Remoteness					< 0.001
Major urban cities	1781 (68.1%)	280 (60.3%)	667 (62.9%)	142 (67.9%)	
Regional or remote area	834 (31.9%)	184 (39.7%)	393 (37.1%)	67 (32.1%)	
Language spoken at home					< 0.001
English	2353 (89.9%)	391 (84.1%)	921 (86.6%)	164 (88.5%)	
Others	29 (1.1%)	15 (3.2%)	39 (3.7%)	3 (1.4%)	
Maternal education					< 0.001
Year 11 or below	437 (16.7%)	103 (22.4%)	314 (30.0%)	93 (45.4%)	
Year 12 or certificate	1292 (49.5%)	216 (47.1%)	565 (53.9%)	84 (41%)	
Bachelor or above	883 (33.8%)	140 (30.5%)	169 (16.1%)	28 (13.7%)	
Low income					< 0.001
No	2028 (77.5)	279 (60.0)	742 (69.8)	118(56.5)	
Yes	590 (22.5)	186 (40)	321 (30.2)	91 (43.5)	
Single parenthood					< 0.001
No	2489 (95.1%)	414 (89.0)	729 (68.6%)	125 (59.8)	
Yes	129 (4.9)	51 (11.0)	334 (31.4)	84 (40.2)	

### Health Inequities (Objective 2)

**Fig. 2:**
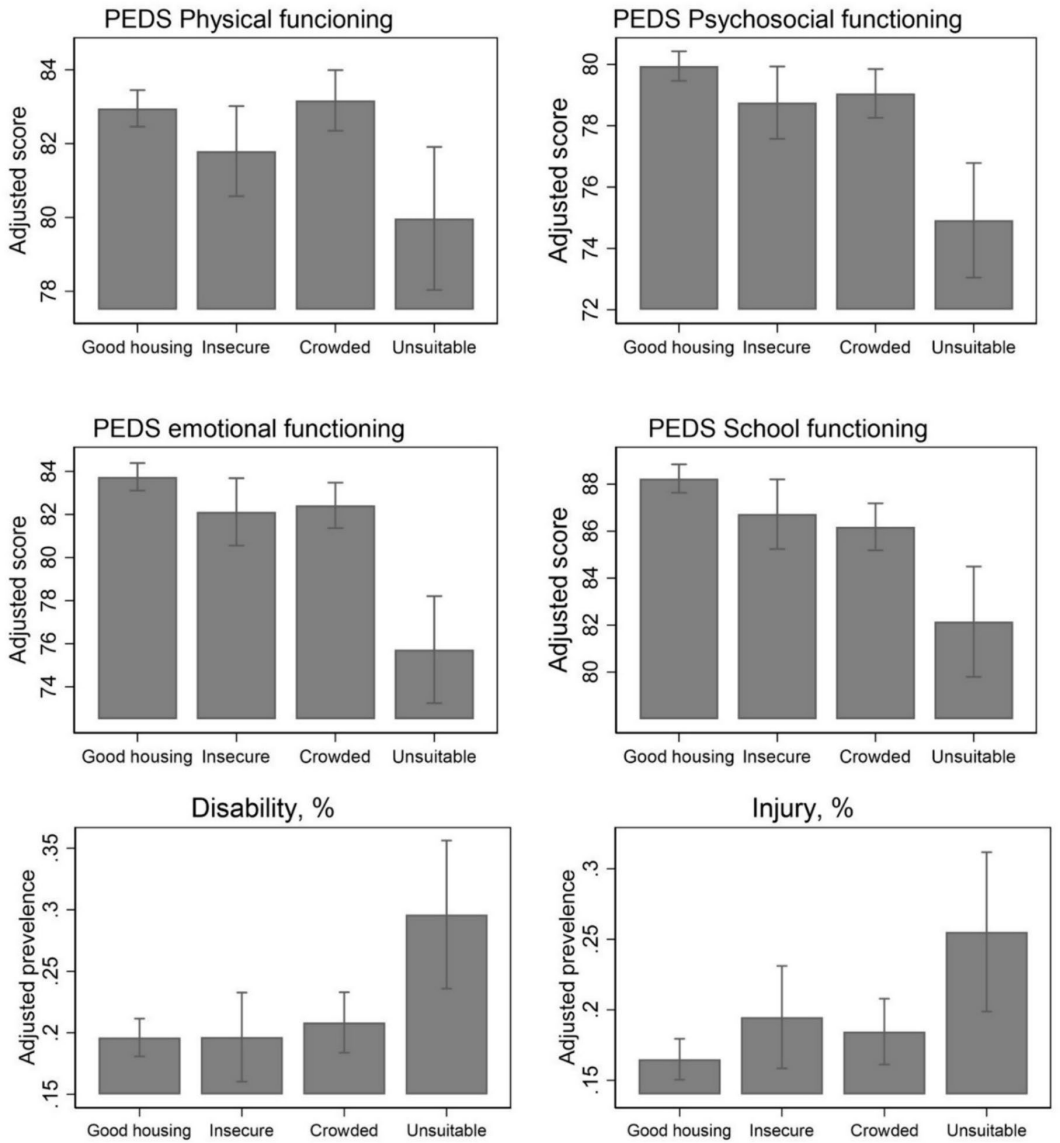
Health and development profile by housing typology, adjusted for children’s age, maternal age, and sample weights (generalized linear regression)

There are noticeable gaps in general developmental indicators, disability, and injury between children in unsuitable housing and the other three typologies (Fig. 2). Children in unsuitable housing had lower scores in physical health (by 3.0-point, 95 CI − 4.99, − 0.98), psychosocial health (by 5.0-point, 95% CI − 6.97, − 3.10), emotional functioning (by 8.0-point, 95 CI − 10.60, − 5.45), and school functioning (by 6.1-point, 95% CI − 8.52, − 3.66), compared to children in good housing (typology 1) (see Supplementary Table 2). Those children in housing disadvantage also have a significantly higher prevalence of disability (PR 1.65, 95% CI 1.25, 2.19), and injury (PR 1.51, 95% CI 1.21, 1.88). Although cross-sectional, health inequities were observed by these patterns in early life.

**Fig. 3:**
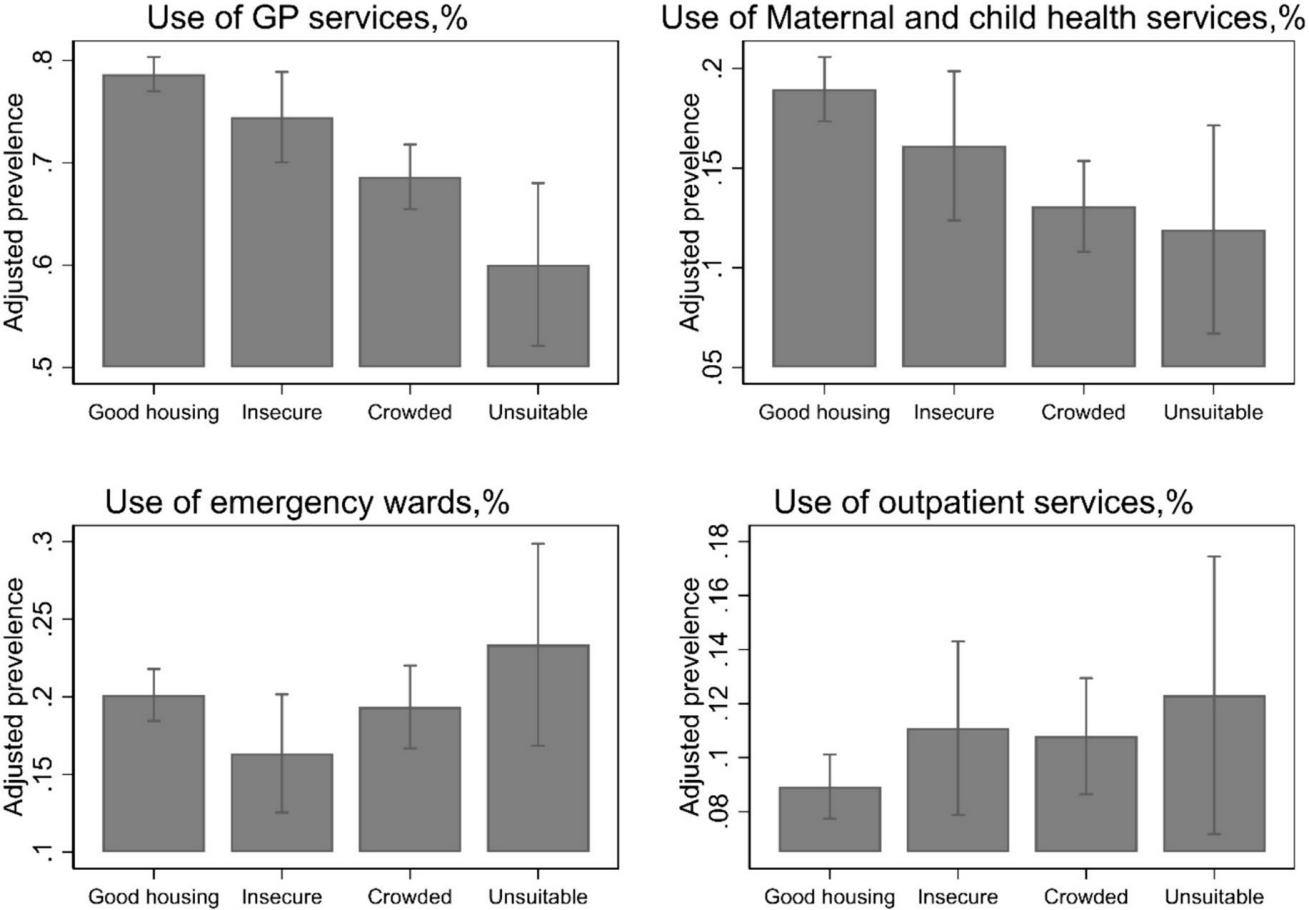
Healthcare use by housing typology, adjusted for children’s age, maternal age, and sample weights (generalized linear regression)

For healthcare utilization, children living in “unsuitable” housing were less likely to use primary healthcare services such as GP visits (PR 0.76; 95% CI 0.67 to 0.87) and more likely to use emergency services (PR 1.16; 95% CI 0.87 to 1.56) and outpatient care (PR 1.38; 95% CI 0.89 to 2.14) (See Supplementary Table 3). This pattern suggests a reliance on emergency services over routine care, potentially due to barriers in accessing primary healthcare (Fig. 3).

## Discussion

We use typologies to describe the housing realities of a nationally representative cohort of Australian preschool-age children. We conceptualize housing disadvantage as a multidimensional construct and use latent class analysis to identify populations exposed to similar types of disadvantages. We then investigated whether these differences in housing experience are associated with health, health service use, and developmental outcomes. In the following section, we discuss the key findings and their implications for policy and clinical practice.

We identified four typologies of housing experienced by this cohort of Australian children. Most children (60%) lived in secure, affordable, and good-condition housing. However, we identified an important group of children (5%) exposed to multiple housing disadvantages, marked by crowding, poor dwelling conditions, and noise—we called this unsuitable housing. This housing disadvantage was matched by financial disadvantages with lone parents, low level of education, and low income being over-represented as living in this category of housing. The four latent classes identified represent qualitatively distinct types of housing conditions, each with unique characteristics and health profiles. These classes do not imply a linear hierarchy of disadvantage but rather different dimensions of housing challenges.

Importantly, we identified gaps in health and developmental indicators between “unsuitable housing” and “good housing” groups. Children in unsuitable housing had significantly worse physical health, psychosocial health, emotional functioning, and school functioning at ages 4 to 5 years. This is concerning because the preschool age is a critical period of child development, and health inequities that emerged during this period are likely to persist across the life course [27]. While our findings do not describe causal associations, evidence of a clustering of disadvantage by housing type supports the use of holistic interventions in early life that encompass the housing, educational, and health sectors.

Our findings have relevance for the clinical care of children. Screening for social determinants of health (e.g., housing) at routine health visits has been officially recommended by medical organizations including the American Academy of Pediatrics [28]. In Australia, there are pilot studies developing screening tools for social determinants of health in a tertiary hospital [29]. While screening tools are relatively well developed for child maltreatment and food insecurity, there is to date no clinically validated tool for housing [29]. This paper sheds light into this by showing that the housing circumstances of the most vulnerable children are characterized by noises, bad conditions, and crowding. More research is needed to further explore the benefits of using these indicators for screening in the clinical setting.

We observed the underutilization of primary healthcare services for children in unsuitable housing. This same subgroup of the cohort is more likely to use emergency rooms and hospital outpatient services and have higher risks of injury and disability. A plausible explanation for the finding is that disadvantaged families delay routine health visits until more severe illness occurs, or that they use emergency services rather than general practitioners because of the cost of gap payments to visit a GP [30]. This finding accords with analyses of maternal and child services in Australia [31]. The underlying mechanisms were conceptualized by the widely recognized health belief model (HBM), that is, individuals’ decisions on preventive health measures (e.g., GP visits) are determined by their perceived susceptibility and severity of disease, perceived benefits and barriers to action, and health motivation [32]. Future research is needed on the integration of housing interventions with health care to achieve equitable use of services.

Several limitations of this study must be noted. First, six out of nine housing variables in the analysis were self-reported, leading to potential misclassification. This may lead to over or underestimation of the prevalence of housing disadvantages. Second, the contribution of certain housing variables (e.g., fuel poverty, unaffordability) to the latent model was small, due to their low prevalence in the sample. Previous literature showed that a prevalence higher than 0.2 would be ideal for binary indicators in latent analysis. However, we opted to retain all indicators in the data to ensure a comprehensive capture of housing disadvantage dimensions. This decision was supported by the overall robustness of our model fit and entropy, indicating good separations between the identified typologies. Third, individuals in subgroup analysis were assigned to latent classes based on the highest posterior probabilities. This widely adopted method, while intuitive, does not incorporate the inherent uncertainty of class membership into its assignment process [33]. We acknowledge this limitation; however, it is important to note that our model’s entropy exceeds 0.7, and the majority (98%) of the sample demonstrated at least a 50% probability of belonging to their assigned class. This indicates that, for our study, the potential loss of information resulting from this assignment method is minimal [34]. Finally, it is important to note the limitations of the LSAC dataset, particularly the underrepresentation of indigenous children in the sample, and the absence of housing measures such as mold and lead paint [15].

This is, to the best of our knowledge, the first study to describe the housing typologies of Australian preschool children. Our findings support the need for collaborations between housing authorities and health systems to screen and identify vulnerable groups and implement targeted interventions in early life to achieve social and health justice.

## Supplementary Information

Below is the link to the electronic supplementary material.

Supplementary file1 (DOCX 16 KB)

## Data Availability

The data of this study is available upon request.
