# Supplementary material for: Housing Australian Children: A Snapshot of Health Inequities in the First 2000 Days
Source: J Urban Health. 2024 Nov 1;101(6):1259–69. doi: 10.1007/s11524-024-00925-0 (PMC11652628; doi:10.1007/s11524-024-00925-0)
Supplement: Supplementary file 1 — Supplementary file1 (DOCX 16 KB) [file 11524_2024_925_MOESM1_ESM.docx]

**Supplementary Table 1. Fit statistics for LCA models of childhood housing disadvantages with 2–7 latent classes**

| **No. of classes** | **G-square** | **AIC** | **BIC** | **aBIC** | **Entropy** |
| --- | --- | --- | --- | --- | --- |
| 2 | 997.64 | 1035.64 | 1156.84 | 1096.47 | 0.56 |
| 3 | 653.55 | 711.55 | 896.54 | 804.39 | 0.75 |
| 4 | 569.60 | 647.60 | 896.39 | 772.46 | 0.71 |
| 5 | 507.63 | 605.63 | 918.20 | 762.50 | 0.65 |
| 6 | 433.72 | 551.72 | 928.09 | 740.61 | 0.78 |
| 7 | 400.84 | 538.84 | 979.00 | 759.75 | 0.78 |

Abbreviations: AIC- Akaike Information Criteria, BIC- Bayesian Information Criteria, aBIC- Adjusted Bayesian Information Criteria

**Supplementary Table 2. Inequalities in health and developmental outcomes by housing typologies, adjusted for children’s gender, maternal age, and sample weights (generalised linear regression)**

|  | Physical health | Psychosocial health | Emotional functioning | School functioning | Injury | Disability |
| --- | --- | --- | --- | --- | --- | --- |
|  | Coefficient (95% CI) | Coefficient (95% CI) | Coefficient (95% CI) | Coefficient (95% CI) | PR (95% CI) | PR (95% CI) |
| Good housing (typology 1) | Reference | Reference | Reference | Reference | Reference | Reference |
| Insecure housing (typology 2) | -1.16 (-2.47, 0.15) | -1.19 (-2.47, 0.08) | -1.63 (-3.32, 0.06) | -1.52 (-3.11, 0.08) | 1.28 (0.99, 1.66) | 1.00 (0.82, 1.22) |
| Crowded housing (typology 3) | 0.21 (-0.75, 1.18) | -0.89 (-1.83, 0.04) | -1.33 (-2.57, -0.08) | -2.05 (-3.23, -0.87) | 1.15 (0.96, 1.38) | 1.06 (0.92, 1.23) |
| Unsuitable housing (typology 4) | -2.98 (-4.99, -0.98) | -5.03(-6.97, -3.1) | -8.03 (-10.6, -5.45) | -6.09 (-8.52, -3.66) | 1.65 (1.25, 2.19) | 1.51 (1.21, 1.88) |

Abbreviations: PR-Prevalence Ratio, CI-Confidence Interval

**Supplementary Table 3. Inequalities in health service use by housing typologies, adjusted for children’s gender, maternal age, and sample weights (generalised linear regression)**

|  | Use of GP services | Use of Maternal and Child health services | Use of emergency wards | Use of outpatient services |
| --- | --- | --- | --- | --- |
|  | PR (95% CI) | PR (95% CI) | PR (95% CI) | PR (95% CI) |
| Good housing (typology 1) | Reference | Reference | Reference | Reference |
| Insecure housing (typology 2) | 0.95 (0.89, 1.01) | 0.85 (0.66, 1.09) | 0.81 (0.64, 1.04) | 1.24 (0.91, 1.70) |
| Crowded housing (typology 3) | 0.87 (0.83, 0.92) | 0.69 (0.57, 0.84) | 0.96 (0.82, 1.13) | 1.21 (0.95, 1.54) |
| Unsuitable housing (typology 4) | 0.76 (0.67, 0.87) | 0.63 (0.40, 0.98) | 1.16 (0.87, 1.56) | 1.38 (0.89, 2.14) |

Abbreviations: PR-Prevalence Ratio, CI-Confidence Interval
